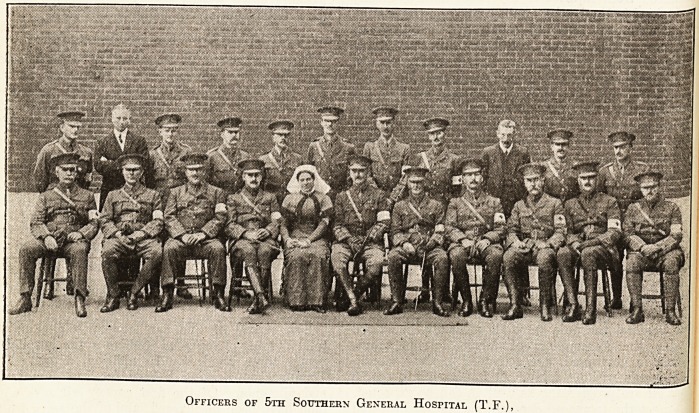# The Southern Territorial Hospital, Portsmouth

**Published:** 1914-10-10

**Authors:** 


### The Southern Territorial Hospital, Portsmouth.

Previous to the outbreak of war, the premises in Fawcett Road, now converted into the 5th Southern Territorial Hospital, Portsmouth, were used as an elementary school. Last month 150 beds were occupied, but the total accommodation amounts to 520. The first batch of wounded, 140 in number, arrived on September 1, and about a fortnight later a further detachment of 54, of whom 14 were Germans, reached the hospital. Like other elementary schools, there is provision for both girls and boys, and at present it is only the beds in the girls' quarters which are in occupation. Their principal schoolroom has been converted into the main ward. The theatre is on the first floor, where the matron's room and store-rooms of various kinds are also situated. The following is a list of the officers:

Lieut.-Colonel John Kyffin, Commanding Officer; Lieut.-Colonel C. P. Childe, F.R.C.S. Eng.; Lieut.-Colonel C. F. Routh, M.D. London; Major T. A. Munro Forde, M.R.C.S., etc.; Major John Phillips, M.D. Edin.; Major E. J. Davis Taylor, M.B., B.C. Camb., Registrar; Major Scott Ridout, M.S., F.R.C.S. Eng.; Major R. Atkinson Dove, M.B.; Captain S. Goss; Captain McEldowney; Captain Lister Wright; Captain H. Burrows, F.R.C.S. Eng., M.B.; Captain C. Lamplough; Captain M. Way; Captain Taylor Morgan; Captain Blackwood; Captain Carling; Captain Lloyd Driver; Captain Dale Wood; Captain P. Green; Lieutenant and Quartermaster L. Blake; and Mr. H. W. Taylor, Honorary Dentist.

Officers of 5th Southern General Hospital (T.F.).

## Figures and Tables

**Figure f1:**